# A spatial method to calculate small-scale fisheries effort in data poor scenarios

**DOI:** 10.1371/journal.pone.0174064

**Published:** 2017-04-13

**Authors:** Andrew Frederick Johnson, Marcia Moreno-Báez, Alfredo Giron-Nava, Julia Corominas, Brad Erisman, Exequiel Ezcurra, Octavio Aburto-Oropeza

**Affiliations:** 1 Marine Biology Research Division, Scripps Institution of Oceanography, La Jolla, CA, United States of America; 2 Centro para la Biodiversidad Marina y la Conservación, La Paz, Mexico; 3 Universitat Politécnica de Catalunya, Barcelona, Spain; 4 Marine Science Institute, University of Texas, Austin, Texas, United States of America; 5 UC MEXUS, University of California Riverside, Riverside, California, United States of America; Department of Agriculture and Water Resources, AUSTRALIA

## Abstract

To gauge the collateral impacts of fishing we must know where fishing boats operate and how much they fish. Although small-scale fisheries land approximately the same amount of fish for human consumption as industrial fleets globally, methods of estimating their fishing effort are comparatively poor. We present an accessible, spatial method of calculating the effort of small-scale fisheries based on two simple measures that are available, or at least easily estimated, in even the most data-poor fisheries: the number of boats and the local coastal human population. We illustrate the method using a small-scale fisheries case study from the Gulf of California, Mexico, and show that our measure of Predicted Fishing Effort (PFE), measured as the number of boats operating in a given area per day adjusted by the number of people in local coastal populations, can accurately predict fisheries landings in the Gulf. Comparing our values of PFE to commercial fishery landings throughout the Gulf also indicates that the current number of small-scale fishing boats in the Gulf is approximately double what is required to land theoretical maximum fish biomass. Our method is fishery-type independent and can be used to quantitatively evaluate the efficacy of growth in small-scale fisheries. This new method provides an important first step towards estimating the fishing effort of small-scale fleets globally.

## Introduction

Global marine fisheries are under pressure from increasing demands for protein, driven by rapidly growing human populations [1,2]. Many commercial stocks remain either fully or over-exploited and show continued declines [3], whilst rules and regulations governing them continue to tighten, driving new fisheries development [4]. Fisheries biomass is often extracted from areas only known by fishers themselves, and is recorded at the point of landing or where the first sale of the catch is made. For this reason, fishing locations often remain elusive, off the radar and difficult to police [5]. Understanding where fish are caught has never been more important. The amount of fishing (the effort) and the area over which fishing potentially takes place (the range) are critical measures for successful fisheries management and making realistic predictions about the wider ecological consequences of fishing [6], although for some specific fishery scenarios this is not always the case [7]. These measures provide important details for management, particularly spatially-explicit approaches such as protected areas or fisheries closures. They also allow more accurate estimates of the economics of fishing regarding time at sea and fuel usage [8,9] and aid in understanding some of the social factors involved in driving fisheries [10].

Landed catch biomass data are now widely recorded for commercial fisheries and are a central component in the design of many fisheries management strategies. Whether or not they are a useful measure of stock status, however, remains undecided [11]. Effort data for fisheries remain comparatively sparse and are often concentrated around the industrial fleet, relying on electronic logbook-ID-type tracking systems such as VMS (Vessel Monitoring System) and AIS (Automatic Identification System) [12]. Methods of calculating fishing effort ultimately depend on the intended applications of such information [13] and different resolutions of data will yield different descriptions of effort [14]. Although this highlights the importance of understanding why such measures are needed, data availability will often be the primary factor determining which measures and calculations can be made [6]. Considering the economic and the multi-national interests often involved, fisheries privacy and data release issues are commonly more of an impediment than the existence of data. Small-scale fisheries often lack data on the number of vessels, gear types used and landing records. This makes the calculation of fishing effort difficult and may bring into question the accuracy of any such results gained for such fleets [13].

The current gap in knowledge for small-scale fishing effort needs addressing if the collateral impacts of such fisheries are to be quantitatively estimated. The importance of understanding these impacts is reiterated when we consider that landings for human consumption from small-scale fisheries are approximately equal to that of the industrial fleet [15]. As McCluskey and Lewison note, *“Small scale does not necessarily mean small impact”* [13]. The approaches that estimate fishing effort for small-scale fisheries are currently labor- and data-intensive, require close cooperation with the fishing sector and involve considerable sampling error and subjectivity, as they do for many data-poor fishery scenarios [16].

The term “effort” encompasses a broad range of meanings and complexities, from nominal measures such as durations of extraction and amounts or sizes of extractive gear to effective measures that standardize effort such as the rate of fish capture or the Catch Per Unit of Effort (CPUE). Such detailed information of a system is often unavailable. For this reason, instead of attempting to design a method that estimates effort based on many case-specific assumptions, we present a new method to estimate fishing effort across broad spatial scales, accounting for data limitations commonly encountered for small-scale fisheries. We did not address temporal scales in the example presented here because of a lack of data, which is a common issue in many other data-poor settings. To illustrate our method we use small-scale fisheries from the Gulf of California, Mexico as a case-study.

We use the term Predicted Fishing Effort (PFE) to describe our new measure, as it estimates the amount of fishing activity (based on the number of boats) within an estimated fishing range. We hypothesize that the human population and the numbers of fishing boats together determine the fishing effort in a given area and the subsequent amount of fish landed locally. PFE is therefore measured as the number of boats per day in an area (in this study a grid cell of 500 km^2^). If PFE estimates are compared to total commercial fisheries landings, our method can also be used to 1) predict the present amount of fish biomass extraction from areas of previously unknown activity; 2) evaluate the potential for future growth in a fishery; and 3) provide an estimate of the most cost-effective number of fishing boats per area without diminishing returns.

## Methods

Unless the relationship between local human populations and fishing boats are perfectly auto-correlated and track each other linearly and normally (i.e. more humans always mean more boats and vice versa), both measures are necessary to calculate a reliable estimate of PFE. By accounting for both measures, areas with high numbers of boats but low numbers of local populations overall (e.g. isolated fishing camps), or areas where there may be low numbers of boats close to extremely high local populations (e.g. major cities with relatively few per-capita fishing vessels) are accounted for.

### Data origin

**Fig 1:**
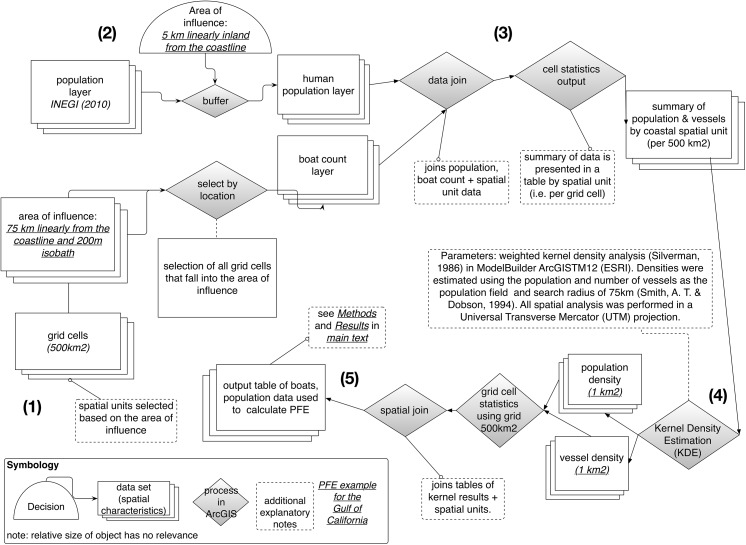
Schematic flow-through diagram describing each stage of the method used to calculate Predicted Fishing Effort (PFE). Method runs clockwise from (1) thru (5).

**Fig 2:**
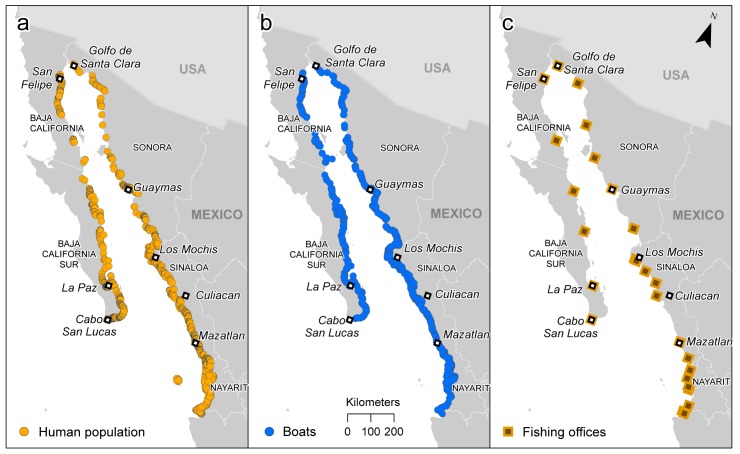
Map of the case-study area, the Gulf of California, used to design the model of Predicted Fishing Effort (PFE). Original locations of raw data used: a) locations at which human population census data were used (*n* = 153 cells), b) locations in which images of boats were counted (*n* = 163 cells), c) location of fishing offices (*n* = 31 cells).

A schematic flow-through diagram illustrating each step in the process of designing and calculating PFE is presented in Fig 1. Human population data were obtained from Mexico’s National Institute of Statistics and Geography (INEGI) [17]. These data were geo-referenced by community. A 5 km buffer inland from the coastline was created to select the majority of the populous within the coastal communities likely to have direct links to the local fishing industry (Fig 2A). The number and location of every coastal in-water small-scale fishing boat in the Gulf was calculated from three different sources: two coastal over-flight surveys conducted by the World Wildlife Fund in 2006 [18] and an estimate using coastal images of the Gulf taken from Google Earth in 2009 [19] **(**Fig 2B). Small-scale boats were defined as any traditional Mexican panga fishing vessel. These are out-board engine-driven, open vessels up to approximately 27 feet (8.2 meters) in length, usually operated by a maximum of 3 crew members with little to no mechanical fishing equipment on board. It is noteworthy that no actual field research was undertaken and therefore no ethics clearance or permits were necessary for data collection. All the data used and produced in these analyses are open source and available from http://datamares.ucsd.edu/eng/projects/fisheries/spatial-distribution-of-potential-fishing-effort-in-the-gulf-of-california-mexico/.

### Spatial analysis and data distribution

There is a substantial difference between the areas fished by the small-scale (coastal) and the industrial (off-shore) fleets within the Gulf of California [20]. The distances travelled by fishers to fishing grounds can therefore vary widely and are determined by factors including target species, time of year, coastline complexity, water depth and weather. The average linear distance travelled by small-scale fishers to their fishing ground in the upper Gulf, was 75 km between 2009 and 2016 [21]. We used this measure to create a buffer delineating the area fished by the small-scale fleet, within which to calculate the PFE. The resultant area that could potentially be fished (herein referred to as the *area of influence*) corresponded well to other published estimates of small-scale vessel activity in the Gulf of California [22]. In ideal scenarios where data are not limited, such areas of influence can be calculated on a case-by-case basis and based on accurate estimates of average areas of activity. Over-conservative buffer areas that are too small will concentrate PFE estimates, whilst buffer areas that are too large will spread PFE estimates too thinly.

To calculate a measure of effort spread throughout the area of influence we created a grid and, within the center of each cell, placed estimated values of the number of boats and human population. We used a conservative grid cell size of 500 km^2^ cells based on the estimated potential fishing area per panga vessel in the Gulf [23]. Only grid cells that intersected with the area of influence were included for further analysis. Values of population and boats were assigned to the centroid of each corresponding cell (herein referred to as the *raw data*). Data on human population size and the number of boats covered a total of 133 grid cells. We used kernel density estimates (KDEs) to spread the data for the human population, the number of boats and the mean total annual catch across the full area of influence (565 grid cells of 500 km^2^). We computed the KDEs with the Spatial Analyst Extension in Model Builder ESRI ArcGIS 10.2 by using the following function [24]:

$$f(u)=\frac{1}{nh}\sum_{i=1}^{n}K(u)$$

Where n represents the total number of input points, *h* represents the search radius (fishing range) of fishers from the departure point (set as 75 km), *K* represents the Kernel function (described below) and *u* represents the input variable (human population, number of boats or total catch (tonnes)). The output of this function has the same units as the input variable, but normalized per unit area (cell-size parameter = 1 km^2^). The Kernel function is described as:

$$K(u)=\frac{3}{4}(1-u^{2})\;|x-x_{i}|\leq h$$

$$K(u)=0\;|x-x_{i}|>h$$

This function describes that for each data point with coordinates *x*_*i*_ from the original dataset, the KDE assigns densities around it according to a quadratic distribution (with a maximum at the original location of the data point) and up to a distance equal to h (75 km) in the interpolated grid with coordinates x for each cell. We transformed the 1 km^2^ resolution data into the 565 grid cells of 500 km^2^ by calculating the mean KDE of each cell (S1 Fig). All the analyses were performed using a Universal Transverse Mercator (UTM) projection.

To build a model of PFE based on human population and the number of boats, we hypothesized that there was a significant relationship between these two variables. To test this hypothesis we ran an Ordinary Least Squares (OLS) regression. This was undertaken for both the raw data (*n* = 133) and the post-KDE data matrix (*n* = 565) to ensure KDE did not alter the relationship between the variables. Significant slope and intercept statistics were then used to build the final PFE model in which PFE was a function of human population and the number of boats. In certain cases or locations, this relationship may not be significant, for example, in very remote or transitory fishing scenarios where the local human population may not relate to the number of boats in the area. To understand if a location may deviate from this linear norm of more population equals more boats, simple regression analyses between the two measures can be used. Outlying cases can either be noted and investigated further, or if justified, excluded from further analysis. In cases with many such non-normal cases, different non-linear regression models may be used to see if a significant relationship between human populations and the number of boats exists. Final significant models should to be incorporated into the PFE estimate using the scaling coefficient of the chosen model (see results for example). If no such relationship is found, then our method should not be used to calculate PFE. We do, however, encourage researchers in data-poor scenarios to persist in their calculations of fishing effort and note that the more that is known about how a fishery and how fishers behaves, the better equipped a team is to investigate ways to accurately estimate fishing effort. The suitability of the human population and boat estimates within areas of influence should be thoroughly evaluated a priori.

### Designing and validating an estimation of effort

#### The relationship between boats and human population

**Fig 3:**
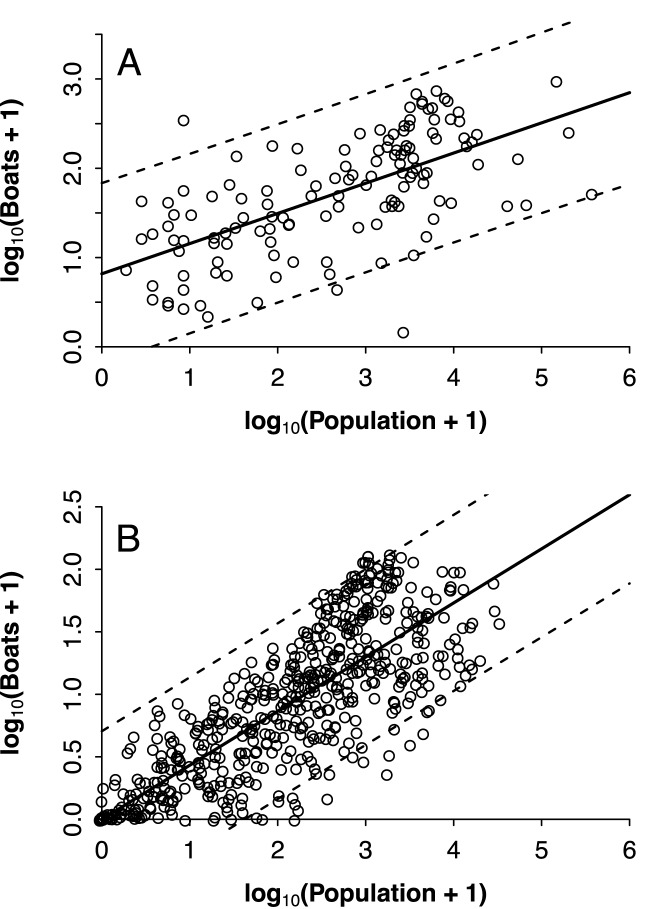
**Relationship between human coastal population and number of small scale fishing boats pre (A) and post (B) kernel density estimation**. Points represent the raw data, solid lines the fitted model and dashed lines the 95% confidence intervals (see also S1A and S1B Fig for mapped post KDE data).

The intensity of fishing depends, in principle, on the demand for fish whether for consumption (local markets) or revenue (non-local markets) purposes. In theory, two variables can consequently be used as indicators of this demand, and hence proxies for PFE: (a) the number of boats in each segment of the coast, and (b) the human population concentrated along the same coastal sector. However, these two variables are in all likelihood related to each other, so they cannot be used as additive predictors of PFE as they may in turn be aliased. We therefore first analyzed how the number of boats scaled-up as the coastal population increases, using allometric equations (or power functions, of the type *boats* = *k*.*population*^*m*^, or, in logarithmic form, log(*boats*) = log(*k*) + *m*. log(*population*)). We found a significant, positive relationship between the logarithm of the number of fishing boats and the logarithm of coastal human population, both pre- (*r*^2^ = 0.40, *F*_1,131_ = 89.51, *p* < 0.001, *m* = 0.34 ± 0.03) and post- (*r*^2^ = 0.65, *F*_1,563_ = 1039, *p* < 0.001) KDE (Fig 3A and 3B), yielding a scaling coefficient *m* = 0.42 ± 0.013 (we added a value of 1 to both the number of boats and the number of inhabitants to avoid the indetermination of log-zero).

The slope for the least squares regression in Fig 3B represents the log-log relationship for the allometric scaling between the number of boats and the population: log(*boats*) = 0.432 × log(*population*). We forced the intercept through the origin given that the absence of people or boats in a location would result in no fishing activity. The equation can be rewritten as log(*boats*) = log(*population*^0.43^). Further, by taking the exponent of both sides, it can be written in a power function form as *boats* = *population*^0.43^. That is, the population-driven number of boats in any sector of the coast can be predicted by the population elevated to an exponent of 0.432.

#### Estimating PFE

The number of boats counted (*boats*) and the number of boats estimated from the population (*population*^0.43^) were linearly related with slope = 1. We therefore combined both variables into a single interaction term to estimate the PFE as the geometric mean of both estimates (*PFE* = (*boats* × *population*^0.43^)^0.5^). Because both estimates are aliased, combining them into a single variable reduces the degrees of freedom of the predictors, making the resulting model more parsimonious. By using a geometric mean, we make sure that extreme cases (high numbers of boats with low coastal populations or vice versa) are accounted for without skewing PFE values. Finally, because in the previous section we showed that *boats* and *population*^0.43^ are linearly related and hence additive in log-form, it follows that in exponent form they should be combined as multipliers. The unit of PFE is therefore the number of boats adjusted by the number of people in local coastal populations. In this case, PFE is calculated using the mean boat count from the three daily snapshots of boat activity in the Gulf. Overall, PFE is measured as the adjusted number of boats per day in a given area (in this case a grid cell of 500km^2^).

#### Validating the estimation of PFE

To validate our measure of PFE we used vessel-tracking data from the upper Gulf of California region. A local collaboration with fishers using hand-held GPS trackers [25] provided us with real travel-tracks of fishing vessels for an average fishing day in the area. From these tracks, we calculated the total number of times fishing vessels fished (see S2 Fig) inside the same grid cell layer that we used for the PFE calculation. We then compared our estimated values of PFE to these real fishing event totals for each grid cell in the upper Gulf of California. OLS linear regression and a Pearson’s correlation of these values were used to determine if a linear relationship between the real and the predicted measures exist and to note the significance of the correlation between the two variables.

### Predicting landings from PFE

We collated annual fisheries data (2001–2013) from the Gulf of California from the Mexican National Commission of Fisheries (CONAPESCA). These data included all small-scale fisheries landings from 31 fishing offices across the whole Gulf (Fig 2C), which collectively register commercial landings from five states: Baja California, Baja California Sur, Sonora, Sinaloa, and Nayarit. These fishing offices are located at major ports and important fishing communities, and provide the most detailed fisheries data available. An independent database that included monthly landings for around 300 species of fish and invertebrates from all 31 offices was obtained from CONAPESCA headquarters in Mazatlan, Sinaloa, Mexico. Fishers are either affiliated to cooperatives or work for private companies that operate based on licenses or concessions granted by CONAPESCA, per resource. All information generated by this federal entity (e.g., human resources, infrastructure, the number of licenses by fishing resource) is included in the National Fisheries Register.

KDE was used to estimate spatially explicit values of mean total annual fisheries landings data (tonnes per 500 km^2^) between 2001 and 2013 for each cell within the area of influence. By comparing PFE to mean total annual landings of all species in the region, we hypothesized that our model of PFE could be used to accurately predict total fisheries landings in the Gulf of California. We tested the relationship with a non-linear least squares regression. It should be noted that because we only consider mean total annual fisheries landings, the PFE estimate is fishery-type independent. If differences between types of fishery need investigation, the method would need to separate different types of fishing boats in the boat counts, calculate a separate PFE for each with fishery-specific assumptions on the range of the fishing boats and separate catch by fishery when comparing predicted landings to PFE estimates.

Fisheries catch is total weight of fish landed, hence the variable is related to fish and/or human demographic processes. In terms of differential calculus, a per capita rate of change is equal to the rate of change of the logarithm of the variable ((1/*y*) d*y*/d*x* = d ln(*y*)/d*x*). We therefore first explored the relationship between PFE and log(catch), to look at the relative change in catch as PFE increases. Once the log model was defined and tested, we transformed it into its arithmetic form to make predictions about expected catch given a certain PFE in any given sector of the coast. The final model (*catch* = *K* * e^-b/PFE^) was fitted to the data using non-linear regression with multiplicative error estimations. To avoid the influence of spatial autocorrelation in our model parameters estimation, we performed 500 random draws of 250 data points each from the PFE and the catch data. For each draw we fitted a logistic curve and estimated K, b and their respective 95% confidence intervals. None of the evaluated fits from the draws showed significant spatial autocorrelation (Mantel test). The final parameters and confidence intervals were calculated as the mean of the 500 estimates generated from the random draws. To approximate normality and homogenize variances, all data was log_10_ (*x*+1) transformed after KDE was applied. All models were run using R (2016) and the *lm* and *nlm* packages.

## Results

We estimated that at any one time there were approximately 17,839 ± 109.42 (standard error of the mean) fishing pangas operating in the coastal waters of the Gulf (between 2006 and 2009). A total of 1,399,951 people lived in coastal communities within 5 km of the coastline in 2010 [17], the majority of them in the 31 cities with fishing offices reporting to CONAPESCA. In 2008, the Mexican National Institute of Statistics reported that 56,113 people (4% of the total coastal population surrounding the Gulf of California) earned profits directly from the fisheries and aquaculture sector in the Gulf of California. Assuming 2 to 3 fishers work per panga, this represents 64% to 95% of this sector.

**Fig 4:**
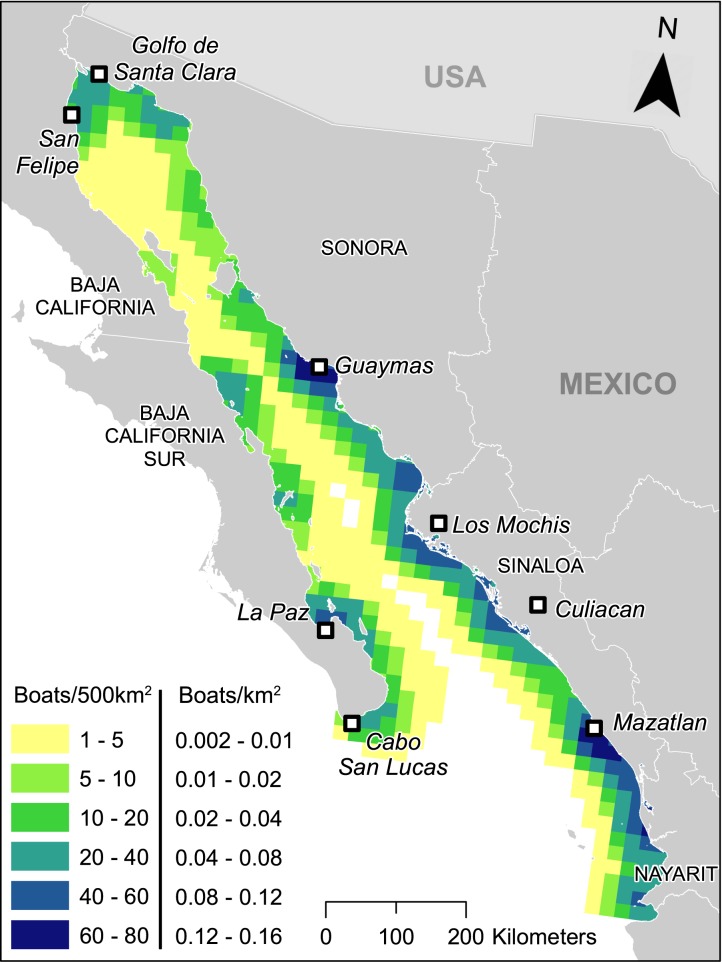
The Predicted Fishing Effort (PFE) presented in 500 km^2^ grid cells (n = 565). The unit of PFE is the number of boats adjusted by the number of people in local coastal populations.

The final PFE estimates for the Gulf of California (Fig 4) suggest that fishing effort was highest close to highly populated human settlements on the coast and/or areas where the number of fishing vessels was high. The highest PFE values spread from the Guaymas (in the north) to Mazatlán (in the south) along the Sonoran and Sinaloan coastline (eastern side of the Gulf of California). La Paz and Cabo San Lucas, the largest cities in this region located towards the south of the Baja California Peninsula, had high values of PFE. San Felipe and Santa Clara in the upper Gulf of California also had high PFE values because of the high numbers of fishing boats in these communities compared with the size of their local populations.

### Does PFE relate to catch?

**Fig 5:**
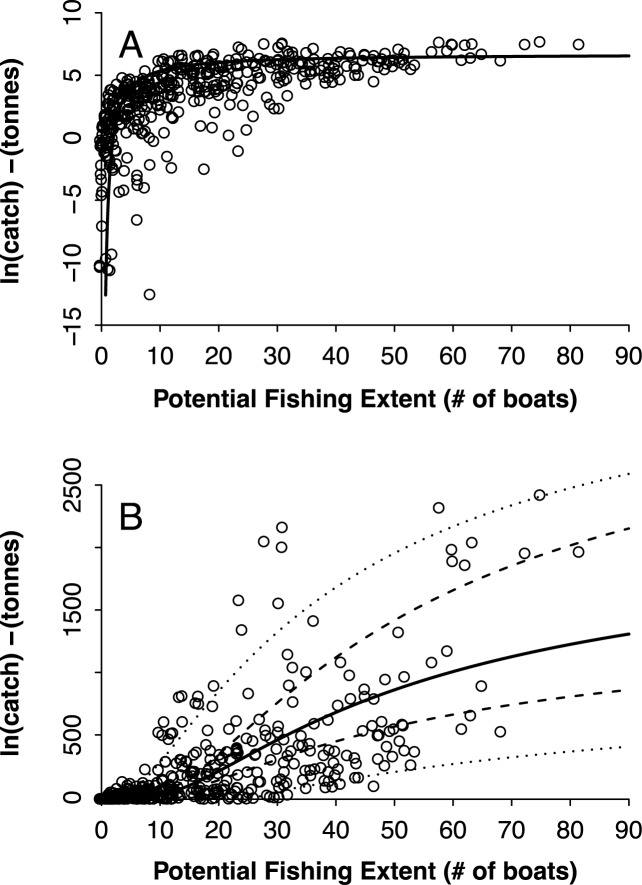
Relationship between Predicted Fishing Effort (# of boats per 500 km^2^) and mean total annual catch. In logarithmic form (A) and arithmetic form (B). Points represent raw data (one value per 500 km^2^ grid cell), solid lines are the fitted non-linear models, the dashed lines are the 95% prediction intervals of the fit, and the outer dotted lines show one standard deviation for the regression residuals. Note the funnel-shaped errors: as PFE increases so does dispersion in the data.

The relationship between PFE and log(*catch*) was asymptotic, in which the relative rate of change (the slope of the fitted trend) decreased as PFE increased, of the type log(*catch*) = log(*K*) –*b*/PFE, where *K* is the maximum total catch and *b* is a scaling or slope parameter describing how fast total catch approaches *K* as PFE increases (Fig 5A). Clearly, as PFE increases total catch approaches *K*, while as PFE decreases total catch approaches zero.

The fit between PFE and mean total annual catch for the arithmetic model was highly significant (*r*^*2*^ = 0.57; *p* < 0.001) with parameter values K = 2204 tonnes per 500 km^2^ per year (95% CI [1451, 3629]) and b = 47.0 (95% CI [31.9, 67.0]; Fig 5B). The fit supports our hypothesis that the use of human population and the number of boats to create a model of PFE can predict fisheries landings in the Gulf of California, whilst accounting for situations that deviate from the linear relationship commonly assumed between local population and the number of boats (small populations with relatively high numbers of boats and large populations with relatively few boats). While PFE has no maximum value (i.e., mathematically it can grow indefinitely), the predicted total catch is bounded between zero and K. The fitted function does not show uniform variances; rather, the variability of the relationship between PFE and total catch increases considerably as PFE grows, exacerbating uncertainty that investing in more boats will increase landings and profits.

The increase in total catch starts to plateau at PFE values of > 23.5 boats per 500km^2^, highlighting the importance of considering K as the maximum total catch per grid cell per year. This inflexion point seen in Fig 5B occurs at total catch values of 298 tonnes per 500 km^2^ per year. After this point, the rate of increase of total catch per number of boats begins to plateau. This suggests that the number of boats at any moment required to reach total catch levels with no further, significant increase in total catch is approximately 23.5 per 500km^2^. Extrapolating this further to the total area of influence (the area we predict is utilized by small-scale fishers in the Gulf = 565 cells = 565*500 = 282,500 km^2^), diminishing catch returns begin when the number of pangas reaches 13,277 at any given moment. This value should, however, be taken with caution as it implies that all areas have the same historical level of fishing. It is much more plausible that different areas have historically different levels of fishing and are therefore at different levels of stock status. On the premise that some of the sites have experienced some level of fishing before our data were collected and are not in a pristine, non-fished condition, the extrapolated value likely overestimates the number of boats required to produce a level of catch at the inflection point of the model.

**Fig 6:**
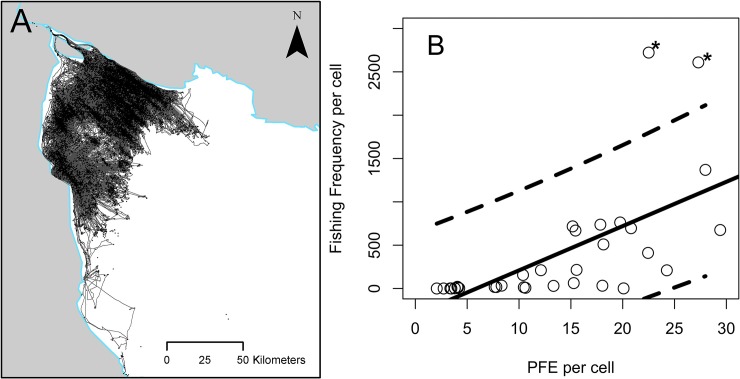
**Map of the upper Gulf of California showing fishing track lines of small-scale fishing vessels (A) and the relationship between PFE per cell and fishing frequency per cell (B)**. Asterisks denote outliers, solid line represents the fitted model and broken lines represent 95% confidence intervals.

It should be noted that we validated the PFE model by testing our PFE values against fishing event values from the upper Gulf of California, where we had access to real tracking information for the small-scale fleet in the area (Fig 6A). OLS regression and a Pearson’s correlation between our estimated PFE values and the real fishing frequency per cell showed a highly significant relationship (*r*^2^ = 0.43; *p* < 0.001, ρ = 0.65, df = 28) (Fig 6B). Two points that lay outside of the 95% prediction limits of the regression were identified as grid cells in which local fishers stop to clean fish on their way back to port, thus replicating the same movement as at the start of a fishing event.

## Discussion

Estimating fishing effort is a necessary requisite for the successful management of marine fisheries. An almost ubiquitous problem with small-scale fisheries is that unless boats are fitted with tracking devices, once they depart on fishing trips, they are for all practical purposes untraceable [13]. By using KDE and the relationship between the local population and the number of boats, we present a new method of estimating the amount of fishing effort within a given area, when supplementary data regarding the fishery are limited or non-existent. In the study area used to build our estimate of fishing effort, PFE was highest in areas of high population density, but also in areas with high numbers of fishing boats relative to local human populations. When incorporating data on locally landed catch, our PFE estimate can also be used to broadly assess the overall efficacy of growth of a small-scale fishery and predict the catch from areas where catch data are unavailable but information on the number of boats and the human population is accessible. Based on our estimates, at any one time in the Gulf of California there were 4,562 more panga vessels operating than the maximum number (13,277) estimated to produce total catches without diminishing returns. We show that the small-scale fishing fleet is at over capacity considering the amount of fish recorded in official catch reports.

Similar methods to the one we present here could also be used to build models that apply to other natural resource extractions that exhibit predictive relationships between local human populations and the number of production units (e.g. boats, diggers, machines etc.) used in the extractive activity, as well as those between the resource extracted and the number of production units measured. Our new method is aimed as a starting point for researchers working in data-poor scenarios tasked with calculating metrics of fishing effort. However, many assumptions and considerations must be acknowledged before the method can be applied in other areas.

### Considerations

The design of our model and the use of the PFE to predict catch and evaluate fisheries growth is based on two significant positive relationships: 1) human population versus the number of boats and 2) the PFE versus the total catch. Satisfying these relationships is a valid assumption for fisheries [16] and to our knowledge there is no literature that suggests a lack of support for the hypothesis that increased coastal human populations generally lead to increased numbers of fishing vessels. An increased catch as a result of increased effort is a common, reasonable assumption which is widely supported until a system is overexploited and a stock begins to collapse [26].

Although our model only requires two simple measures, how these are derived may have a significant bearing on the outputs from the method. For example, the accuracy of estimates for the number of boats is likely to vary widely depending on how such boat-count data are collected. In our example, we benefited from aerial survey estimates, which are unlikely to be available in many small-scale fishery scenarios. Observer data for moored boats and boats within site of land are the most probable methods of boat number estimation in many cases. We believe that using additional, reliably sourced data to calculate mean values is a more robust method than using single year data values. For this reason, we calculated a mean of 3 estimates of the number of boats spanning 4 years, as well as more than a decade of catch data to estimate the total mean annual catch per unit area (tonnes / 500km^2^ / year). Our final PFE estimates are therefore a better general reflection of fishing effort and its relationship to mean annual total landed catch per area for the Gulf than if we were to rely on single-value estimates. If increased temporal accuracy is required for an effort estimate, it will be necessary to collect data on fishing boats, human population and biomass landed within the time period for which estimates are required. The accuracy and potential error associated with each of these variables, and the amount of fish landed should still, however, always be thoroughly acknowledged.

The method we present assumes a reasonable knowledge of the mean distance travelled by fishers during an average trip and the extent of local human communities likely to be closely involved with local small-scale fishing fleets. Our validation method benefited from high resolution, voluntary tracking data supplied by fishers [21] and the isolated and discrete coastal communities along the Baja California peninsula. Such a scenario is rare, but approximate estimates of distances travelled by fishers can be calculated by time at sea in conjunction with additional knowledge of fishing behavior and vessel range, perhaps gleaned through simple questionnaires [27]. It is important to note that by extrapolating our validation from a specific location (the Upper Gulf) to the whole Gulf, we missed potential differences in the productivity or fishing activity of different areas. An ideal scenario would be to have multiple sets of tracking data from all over the Gulf. This, however was not available and, as with much of our approach, the idea was to make a best attempt considering such data-limited scenarios.

As a model estimate, our PFE should be applied and interpreted with a degree of caution based on the aforementioned assumptions as well as the following limitations. The method does not account for protected areas. Protected areas, however, can easily be demarcated when defining the overall area of influence. Additionally, only using small-scale boats in our calculation of PFE excluded industrial fisheries from our estimates. Conclusions made regarding the collateral impacts of local fisheries should only be drawn for the fleet for which boat estimates were taken and should acknowledge activity from those vessels not included in boat number estimates built into the primary model. Addressing these limitations and assumptions is an area that will likely prove fruitful in further developing the method to increase its accuracy and reliability. The method will also benefit from more road testing in places with different types of fisheries, economic strategies and regulatory environments. Having said this, we believe that our PFE estimates for the Gulf of California accurately captures the effort of the small-scale fleet [28,29].

### Fishing effort in the Gulf of California

By building a model to estimate PFE we were able to estimate and visualize where small-scale fishers are most active in the Gulf of California. Comparison of PFE estimates to total landed catch data showed that future investment in the Gulf’s fisheries may not be economically or ecologically viable. Assuming consistent landed values of fish, the catch plateau at around 23.5 boats per 500 km^2^ per day and indicated there may be little economic sense in adding more boats close to, or past this point in many areas of the Gulf. In areas with PFE values over 23.5, economic and ecological maxima may have already been reached. Increasing fishing in these areas will likely yield minimal additional landings while increasing economic costs and damage to exploited stocks and local ecosystems. This means that more biomass per grid cell is being removed than we built into the model. Fisheries in the areas of the Gulf with high PFE values are therefore likely to underperform, like many other global fisheries. In total, we estimate that 17,839 small-scale boats operated at any given moment in the Gulf between 2006 and 2009, 34% more than would produce maximum total catch without diminished returns. This value increases to 88%, almost double over capacity, when considering more recent estimates (2010) of total boats operating (25,000 boats approx. [30]). We therefore clearly demonstrated overcapacity in the Gulf’s small-scale fishing fleet. Our estimates are also highly conservative as they do not account for a growing fleet [30], illegal or unreported fisheries, bycatch or the ecosystem impacts of the commercial fleet, all of which will affect standing stock biomass. Future fisheries growth in the Gulf of California should therefore be addressed very carefully with a long-term outlook that accounts for ecological sustainability, economic efficiency and the effort of the local fishing fleets.

## Conclusions

We have presented a new method that allows small-scale fisheries effort to be estimated based on simple measures attainable, in even the most data-poor scenarios. If the same step-by-step methodology is used, all assumptions backed by robust data and limitations acknowledged, this method will facilitate predicting fishing effort for other data-poor fisheries. Estimates of human population are now commonplace and easily attainable as are estimates of landed catch in FAO-type databases [31], albeit at regional and national scales. We foresee the main data limitation coming from estimates of the number of fishing boats present in a given area. New, high-resolution satellite technologies appear the best way to address this issue in areas where accurate estimates may be difficult to otherwise calculate. When images from these technologies become easily accessible and available in all areas, the challenge will then be to source workforces or automated methods to make counts of the boats. Resultant effort estimates will prove important in highlighting areas that experience high fisheries impacts, and places that may be candidates for protection. Although we do not suggest that our model is exhaustive and fully incorporates all variability in natural and human systems, we believe it is a robust and valid start to elucidating the fishing effort of small-scale fisheries. This new method of effort estimation moves us away from data-intensive and laborious methods of investigating this important measure. Our method predicts the ecological impacts of increasingly concentrated fishing efforts, as well as the economic potential for future expansion. We hope that our PFE method can shed some light on the effects of small-scale fisheries globally. This will benefit management and conservation decisions if presented objectively and in a timely manner, particularly in data-poor scenarios.

## Supporting information

S1 Fig Map of the case-study area, the Gulf of California, used to design the model of Predicted Fishing Effort (PFE). A) Human population data (raw n = 153) and B) vessel density data (raw n = 163) following Kernel Density Estimation spatial extrapolation presented in 500 km^2^ grid cells (n = 565). BC = Baja California, BCS = Baja California Sur, SON = Sonora, SIN = Sinaloa, and NAY = Nayarit.
(TIF)

S2 Fig **Classification of point data generated by a GPS data-logger** using the speed to identify where fishing gear is potentially deployed and retrieved in the upper gulf of California for (A) gillnets and (B) trawlers. An example of a fishing event is highlighted in the zoomed boxes.
(DOCX)
