# Correction: A spatial method to calculate small-scale fisheries effort in data poor scenarios

**DOI:** 10.1371/journal.pone.0179114

**Published:** 2017-06-01

**Authors:** Andrew Frederick Johnson, Marcia Moreno-Báez, Alfredo Giron-Nava, Julia Corominas, Brad Erisman, Exequiel Ezcurra, Octavio Aburto-Oropeza

Fig 5 appears incorrectly. Please see the corrected Fig 5 here.

**Fig 5:**
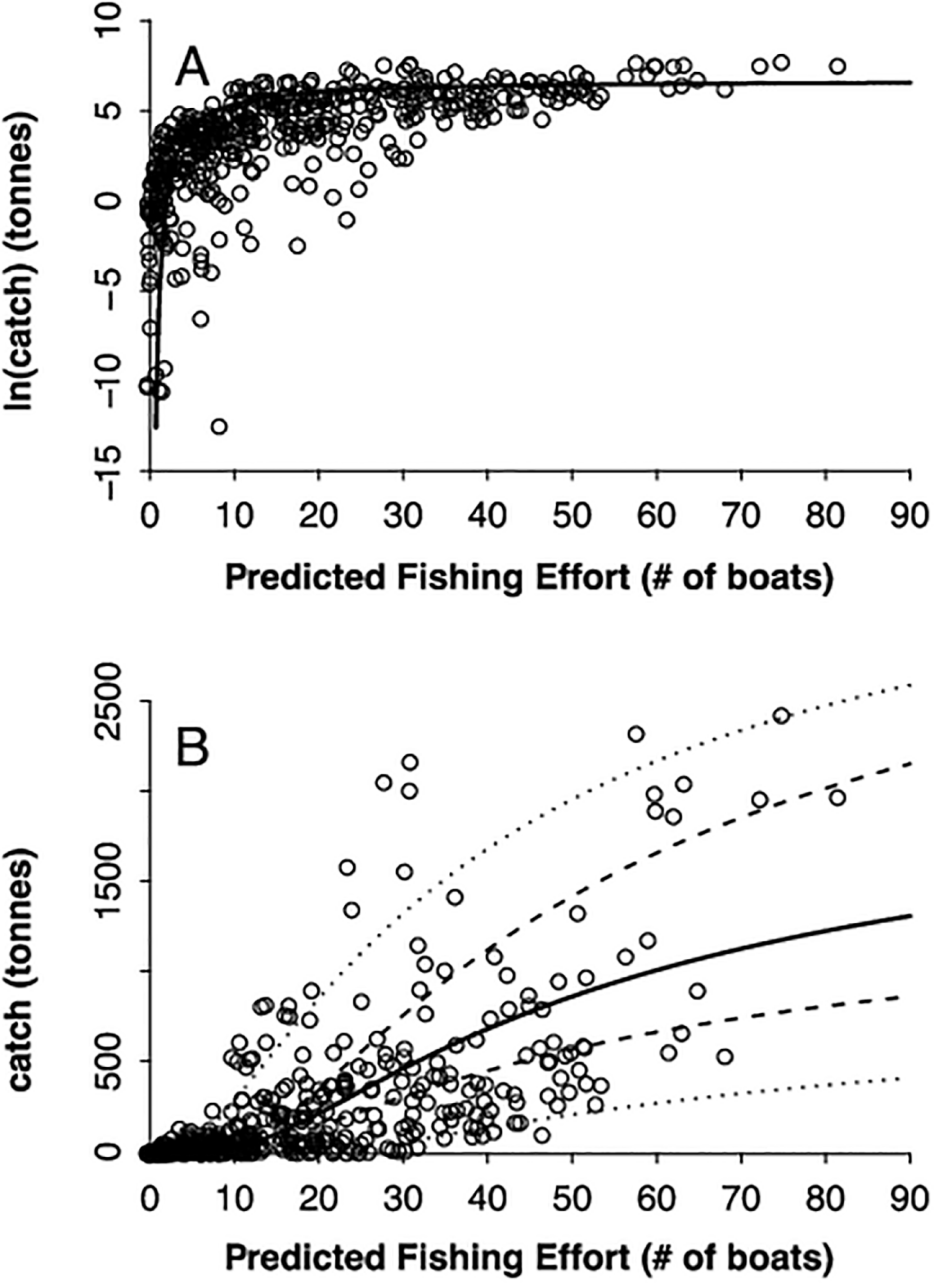
Relationship between predicted fishing effort (# of boats per 500 km^2^) and mean total annual catch. In logarithmic form (A) and arithmetic form (B). Points represent raw data (one value per 500 km^2^ grid cell), solid lines are the fitted non-linear models, the dashed lines are the 95% prediction intervals of the fit, and the outer dotted lines show one standard deviation for the regression residuals. Note the funnel-shaped errors: as PFE increases so does dispersion in the data.
