# Supplementary figures and images for: A spatial method to calculate small-scale fisheries effort in data poor scenarios
Source: PLoS One. 2017 Apr 13;12(4):e0174064. doi: 10.1371/journal.pone.0174064 (PMC5390979; doi:10.1371/journal.pone.0174064)

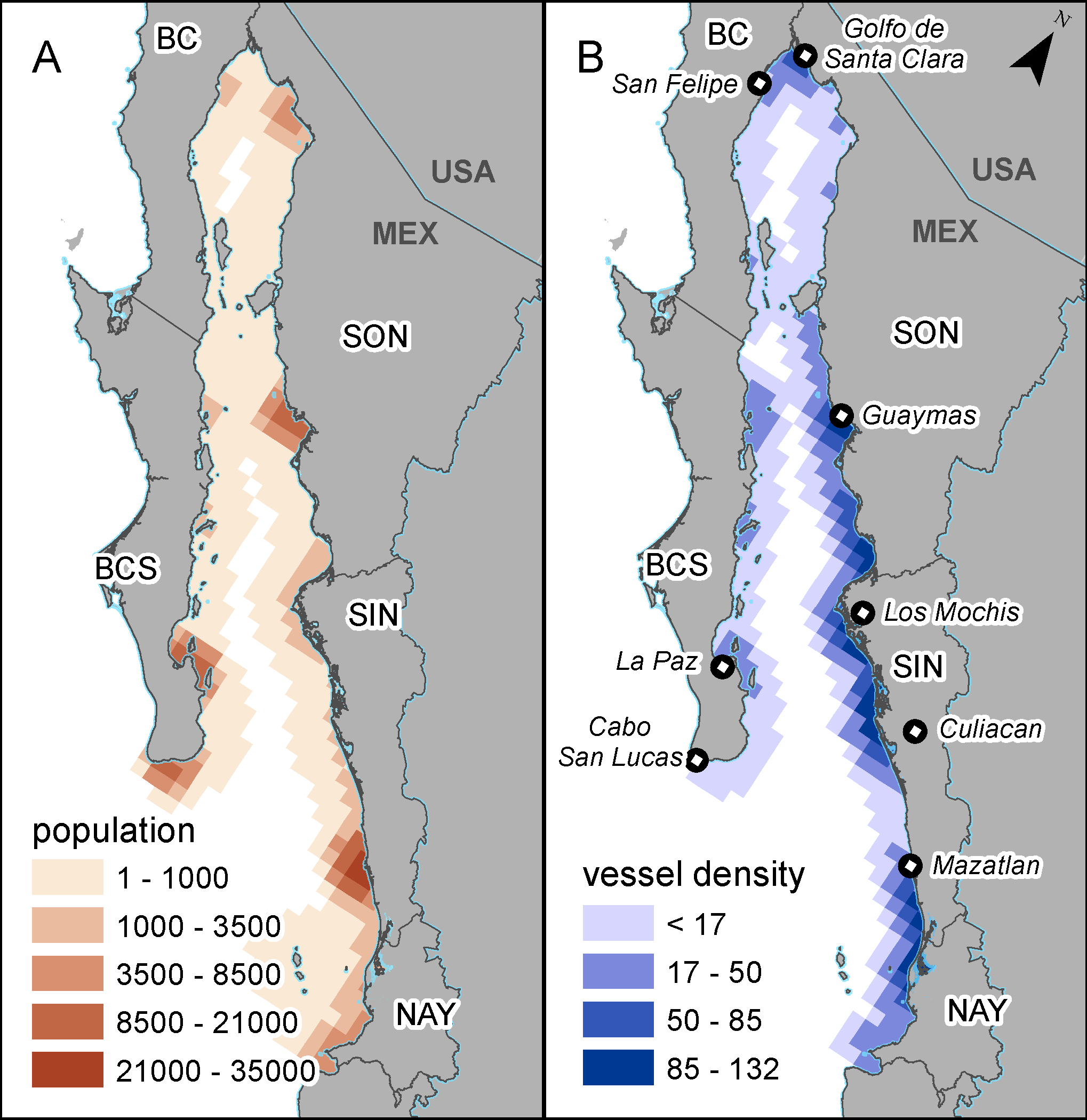

Supplement: S1 Fig — A) Human population data (raw n = 153) and B) vessel density data (raw n = 163) following Kernel Density Estimation spatial extrapolation presented in 500 km2 grid cells (n = 565). BC = Baja California, BCS = Baja California Sur, SON = Sonora, SIN = Sinaloa, and NAY = Nayarit. (TIF) [file pone.0174064.s001.tif]
