# Supplementary material for: A spatial method to calculate small-scale fisheries effort in data poor scenarios
Source: PLoS One. 2017 Apr 13;12(4):e0174064. doi: 10.1371/journal.pone.0174064 (PMC5390979; doi:10.1371/journal.pone.0174064)
Supplement: S2 Fig — Classification of point data generated by a GPS data-logger using the speed to identify where fishing gear is potentially deployed and retrieved in the upper gulf of California for (A) gillnets and (B) trawlers. An example of a fishing event is highlighted in the zoomed boxes. (DOCX) [file pone.0174064.s002.docx]

Supplementary Material Appendix 2

**Fishing Zones Identification and Analysis**

Data was transformed into vector features for initial analysis and visualization. The track data was visualized directly in ARC GIS when imported and converted to a CSV (comma separated value) file format, consisting of x-coordinates and y-coordinates to facilitate its use and its integration into a GIS. By using the speed and vessel maneuvers we were able to identify where the gear was deployed and retrieved during a trip event (Figure S1); fishing gear is deployed shortly after the boat has come to a stop and starts to drift with the tide.The analysis was performed in a Universal Transverse Mercator (UTM) projection. ESRI ArcGIS 10.1 was used for all spatial analysis.

We used a 500m x 500m grid size to calculate frequency by means of spatial union. The spatial degree of use by vessels within each spatial polygon was estimated based on the times of fishing presence inside any particular grid cell (500km^2^ cells) and it was performed with the summarize tool in ArcGISTM 10.1.


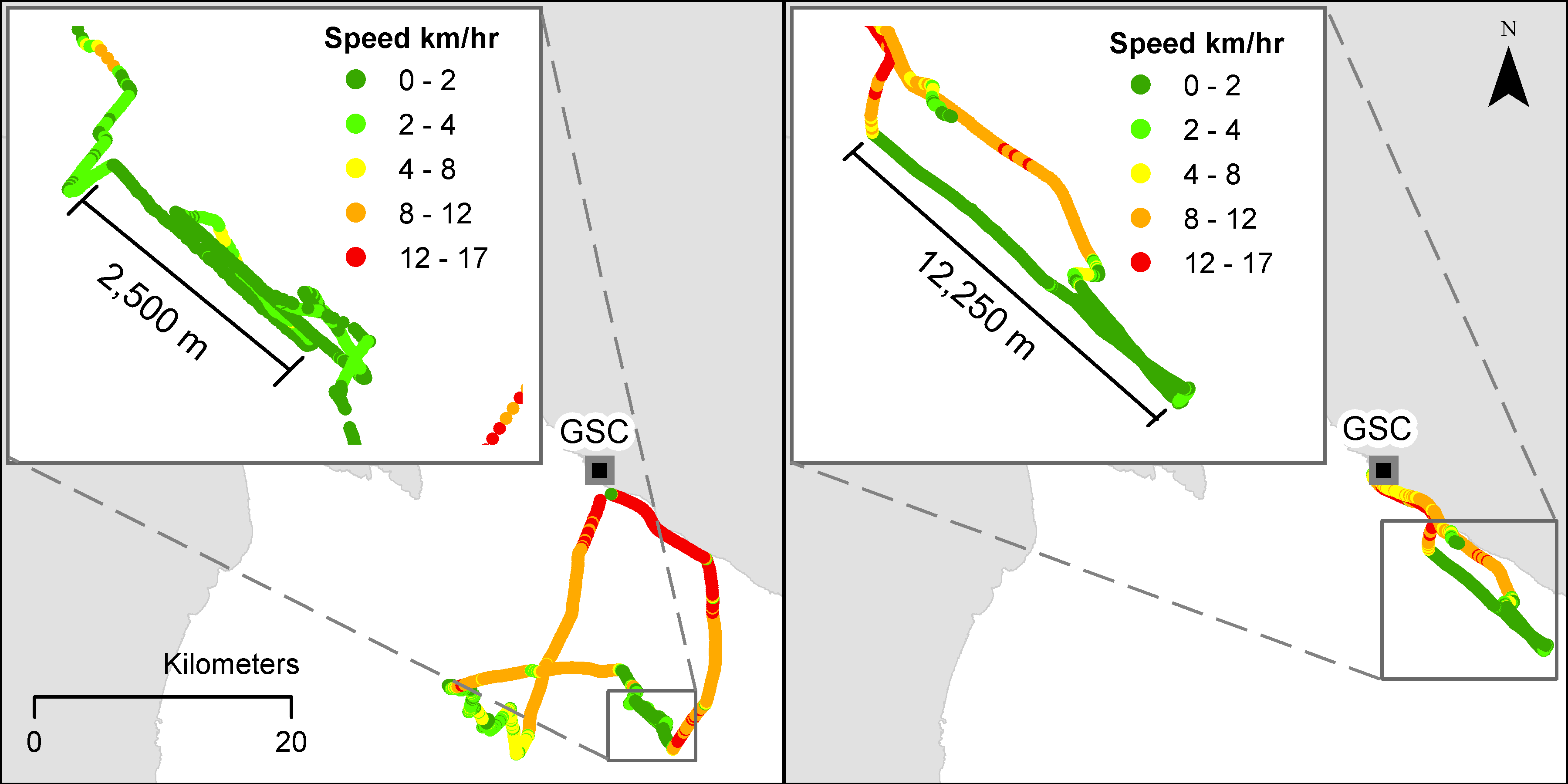


(A)

(B)

S2.Figure 1. Classification of point data generated by a GPS data-logger using the speed to identify where fishing gear is potentially deployed and retrieved in the upper gulf of California for (A) gillnets and (B) trawlers. An example of a fishing event is highlighted in the zoomed boxes.
